# A Genome-Wide Analysis of the CEP Gene Family in Cotton and a Functional Study of *GhCEP46-D05* in Plant Development

**DOI:** 10.3390/ijms25084231

**Published:** 2024-04-11

**Authors:** Zhenyu Mei, Bei Li, Shouhong Zhu, Yan Li, Jinbo Yao, Jingwen Pan, Yongshan Zhang, Wei Chen

**Affiliations:** 1 National Key Laboratory of Cotton Bio-Breeding and Integrated Utilization, Institute of Cotton Research, Chinese Academy of Agricultural Sciences, Anyang 455000, China; 2 Zhengzhou Research Base, National Key Laboratory of Cotton Bio-Breeding and Integrated Utilization, School of Agricultural Sciences, Zhengzhou University, Zhengzhou 450001, China

**Keywords:** CEP, cotton, genome-wide analysis, expression pattern, functional study

## Abstract

*C-TERMINALLY ENCODED PEPTIDEs* (*CEPs*) are a class of peptide hormones that have been shown in previous studies to play an important role in regulating the development and response to abiotic stress in model plants. However, their role in cotton is not well understood. In this study, we identified 54, 59, 34, and 35 CEP genes from *Gossypium hirsutum* (2n = 4x = 52, AD1), *G. barbadense* (AD2), *G. arboreum* (2n = 2X = 26, A2), and *G. raimondii* (2n = 2X = 26, D5), respectively. Sequence alignment and phylogenetic analyses indicate that cotton CEP proteins can be categorized into two subgroups based on the differentiation of their CEP domain. Chromosomal distribution and collinearity analyses show that most of the cotton CEP genes are situated in gene clusters, suggesting that segmental duplication may be a critical factor in CEP gene expansion. Expression pattern analyses showed that cotton CEP genes are widely expressed throughout the plant, with some genes exhibiting specific expression patterns. Ectopic expression of *GhCEP46-D05* in *Arabidopsis* led to a significant reduction in both root length and seed size, resulting in a dwarf phenotype. Similarly, overexpression of *GhCEP46-D05* in cotton resulted in reduced internode length and plant height. These findings provide a foundation for further investigation into the function of cotton CEP genes and their potential role in cotton breeding.

## 1. Introduction

Phytopeptide hormones play an essential role in regulating plant growth, development and adaptation in a non-cell-autonomous manner through intercellular communication [1,2,3]. The C-TERMINALLY ENCODED PEPTIDE (CEP) family is one of the most universal peptide classes [4]. The first CEP gene identified was *AtCEP1*, which was discovered using an in silico approach in *Arabidopsis thaliana* [5]. CEP genes are abundant in seed plants, but not in ancient land plant lineages that lack true branching roots or root vasculature [6]. To date, 16 members of the CEP family have been identified in *Arabidopsis thaliana* [6,7]. Each CEP consists of a signal peptide at the N-terminus and a certain number of conserved CEP domains at the C-terminus. In *Arabidopsis*, members of the CEP family have been shown to have distinct expression patterns during development, and the expression of some CEP genes could be regulated by environmental cues such as exogenous hormones, nutrients and stress. 

Recent evidence has implicated CEPs in the regulation of plant development and response to abiotic stress in model plants [8]. For example, constitutive expression of *AtCEP1* in *Arabidopsis* suppressed primary root growth and delayed lateral root elongation by inhibiting meristematic cell division and expansion [5,9]. In *AtCEP3*-overexpressing plants, primary root length was significantly reduced but shoots were enlarged, fewer rosette leaves of reduced size were differentiated, and flowering time was delayed. In addition, leaf morphology was altered (more flattened and rounded). *AtCEP3* could be induced by nitrogen-limiting or high-salt conditions, and *cep3* knockout lines showed increased root system size under these abiotic stress conditions [7,10]. Overexpression of *OsCEP6.1*, a CEP gene highly expressed in reproductive tissues in rice (*Oryza sativa*), causes multiple phenotypic variations, such as reduced primary root length and seedling height, fewer tillers, shorter panicle lengths and smaller flag leaves [11]. In addition, several rice CEP genes were found to be reduced by various abiotic stresses, and abiotic stress-responsive elements were enriched in their promoters [12]. *ZmCEP1*, a homolog of *OsCEP6.1* in maize, is preferentially expressed in reproductive organs such as young ears and tassels. Overexpression of *ZmCEP1* reduces plant height, spikelet size and kernel size. Kernel length and width are significantly increased in *ZmCEP1* knockout lines, and plant and ear height are also slightly increased [13]. In *Setaria italica*, 14 CEP genes have been identified whose expression can be induced by various abiotic stresses (such as salt and drought) and phytohormones [such as abscisic acid (ABA)]. One of them, *SiCEP3*, was highly expressed in panicles. Overexpression of *SiCEP3* inhibits plant growth, and exogenous synthetic SiCEP3 promotes ABA signalling by increasing ABA uptake [4,14]. Post-translational modification of CEPs also affects their activities. For example, in *Medicago truncatula*, overexpression of *MtCEP1* inhibits lateral root formation but promotes nodulation. MtCEP1 with different hydroxylation patterns was found to exhibit differential inhibition of lateral root formation and altered patterns of auxin response [15]. 

Several components of the CEP signalling cascades have been identified, some of which are also involved in the signalling of other hormones. In *Arabidopsis*, two leucine-rich repeat receptor kinase (LRR-RK) genes have been identified as CEP receptors (*CEPR1* and *CEPR2*). The CEP genes are induced by nitrogen starvation, and CEP peptides secreted in the root may act as long-distance root-to-shoot signals received by these two receptors [16]. CEPR2 can interact with and phosphorylate NITRATE TRANSPORTER 1.2 (NRT1.2), an ABA transporter with ABA import activity that positively regulates the ABA response [17]. In addition, CEPR2 interacts with and phosphorylates some members of the pyrabactin resistance 1 (PYR1)/PYR1-like (PYL) abscisic acid (ABA) receptors [18]. The *cepr1* knockout mutants showed a reduction in vegetative and reproductive growth, and subsequently, seed yield and seed size were significantly reduced [19]. In *Medicago truncatula*, *COMPACT ROOT ARCHITECTURE2* (*CRA2*), a homologue of *CEPR1*, is a receptor of *MtCEP1*. CRA2 interacts with MtCEP1 to regulate ethylene signalling [20]. Thus, CEP signalling usually shows crosstalk with other hormone signals.

Cotton is one of the most important fibre crops in China and the world. The improvement of cotton yield and quality is highly desired for cotton breeding [21]. Although CEPs have been shown to be important phytopeptide hormones that modulate plant or stress resistance in model plants, the role and function of CEP genes in cotton are still poorly understood. Identification and analysis of the phylogenetic relationships of CEP genes in cotton, their chromosomal locations, domain structures, collinearity, expression patterns and functions in cotton development would benefit the understanding of CEP genes and provide important gene resources for yield improvement in cotton. In this study, we performed a comprehensive analysis of CEP genes in four cotton (*Gossypium*) species (*G. hirsutum*, *G. barbadense*, *G. raimondii* and *G. arboretum*) and a functional study of *GhCEP46-D05* was conducted to explore the potential role of cotton CEP genes in plant development.

## 2. Results 

### 2.1. Genome-Wide Identification of CEP Genes

Using the amino acid sequences of 16 reported CEP proteins from *Arabidopsis* as queries in BLASTP, 46, 44, 57, and 72 candidate CEP genes were identified from *G. arboreum*, *G. raimondii*, *G. hirsutum* and *G. barbadense*, respectively. After filtering for the presence of signal peptides and CEP domains, 34, 35, 54 and 59 CEP genes were retained, respectively. In each genome, the identified CEP genes were renamed according to their chromosomal locations, with suffixes added to represent the chromosomes (*GaCEP1-01~GaCEP34-14*, *GrCEP1-02~GrCEP35-13*, *GhCEP1-A01~GhCEP54-D13* and *GbCEP1-A01~GbCEP59-D13*).

The basic characteristics of the cotton CEP genes, such as gene length, exon number, molecular weight and isoelectric point of the protein, are listed in Table S1. Most of the cotton CEP genes have only one exon, and the others contain 2–5 exons. Most of the cotton CEPs encode small proteins (77–214 amino acids; 7.09–21.4 kDa), except for GbCEP2-A01 (546aa), GbCEP37-D03 (391aa) and GhCEP37-D03 (410aa). The theoretical isoelectric points and charges of the cotton CEP genes show a wide range of variation, with theoretical isoelectric points ranging from 4.45 to 11.48 and charges from −8.5 to 17.5.

### 2.2. CEP Gene Structure and Phylogenetic Tree Analysis

**Figure 1:**
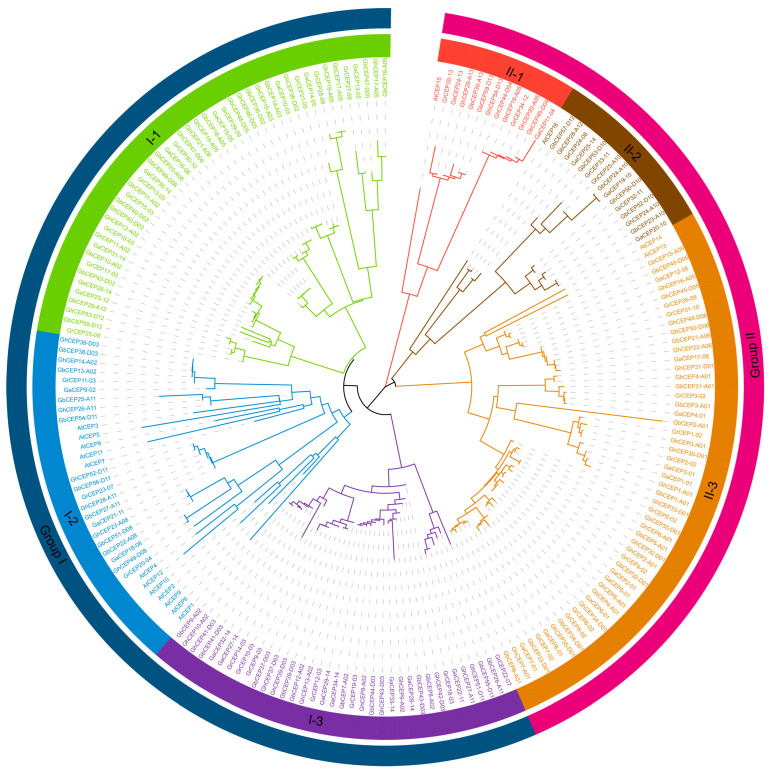
Phylogenetic relationships of CEP genes in *G. arboreum* (Ga), *G. raimondii* (Gr), *G. hirsutum* (Gh), *G. barbadense* (Gb), and *Arabidopsis* (At). A neighbour-joining (NJ) phylogenetic tree was constructed in MEGA X based on full-length protein sequences of CEP genes.

A phylogenetic analysis was performed using the complete protein sequences of *Arabidopsis* and cotton CEPs to construct a tree. Consistent with previous research, the resulting phylogenetic tree was separated into two distinct groups: Group I and Group II (see Figure 1 for details). Each group could be further subdivided into three subgroups. In Group I, all Arabidopsis CEP genes (*AtCEP1*–*AtCEP12*) were located in subgroup I-2, and subgroups I-1 and I-3 contained only cotton CEPs. Group II contained four Arabidopsis CEP genes (*AtCEP13*–*AtCEP16*); subgroup II-1 and subgroup II-3 each contained one Arabidopsis CEP gene (*AtCEP16* and *AtCEP15*, respectively).

**Figure 2:**
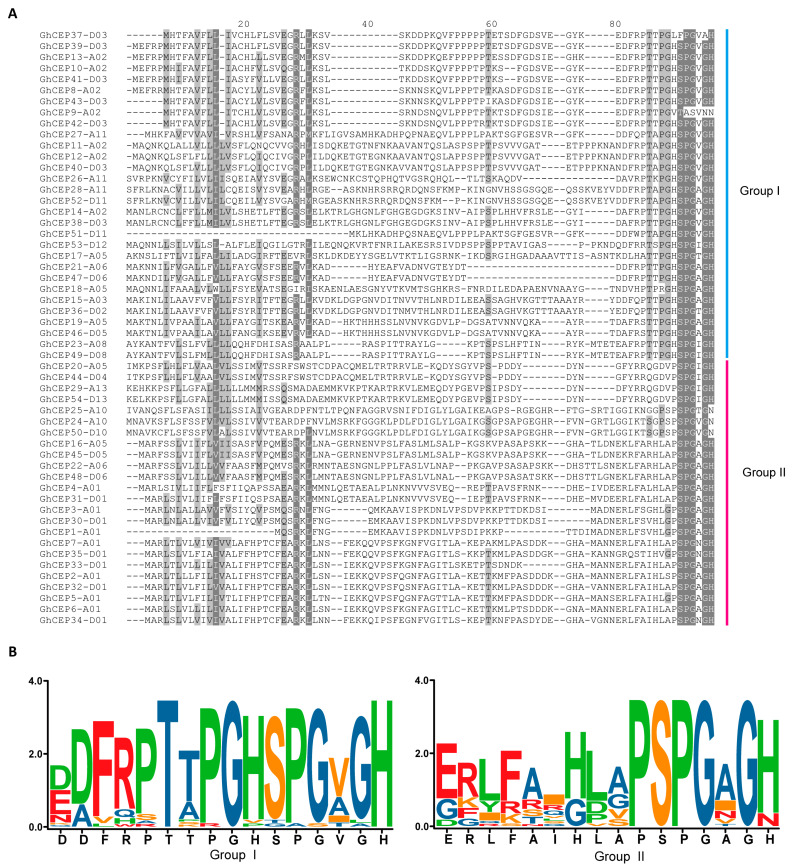
CEP domains of the cotton CEP genes. (**A**) Alignment of the CEP domains of *G. hirsutum* CEP genes. (**B**) Sequence logos of CEP domains in *G. hirsutum*.

WebLogo plots showed that the CEP domains of the cotton CEP genes had a high amino acid similarity between two groups and that a core motif (SPG [V/A/I] G [H/N] in Group I and PSPG [V/A/I] G [H/N] in Group II) was present in both groups. Another motif (PT [T/A] PGH) was also found in front of the core motif in Group I (Figure 2). Thus, the CEP domains contain three prolines in Group I and two prolines in Group II. Previous studies have shown that hydroxyprolylation can affect the biological activity and hydrophilicity of CEP peptides [5,15].

### 2.3. Chromosomal Location and Collinearity Analysis of the CEP Gene in Cotton

**Figure 3:**
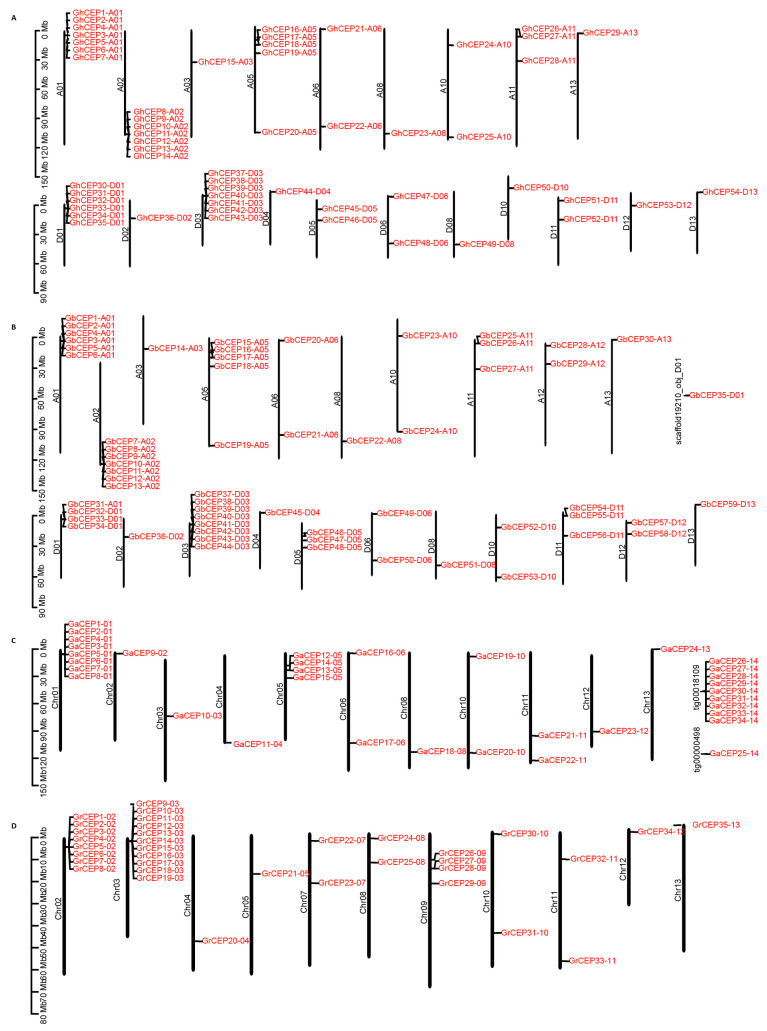
Distribution of cotton CEP genes in: (**A**) Gh. (**B**) Gb. (**C**) Ga. (**D**) Gr.

The distribution of CEP genes is relatively scattered across the genome, with most of the chromosomes in the four cotton species harbouring CEPs (Figure 3). In *G. arboreum*, 34 CEP genes are located on 11 chromosomes, and the rest are located on two scaffolds. In the *G. raimondii* genome, a total of 35 CEP genes are found on all chromosomes, except Chr01 and Chr06. In *G. hirsutum* and *G. barbadense*, the distribution of CEP genes spans 20 and 21 chromosomes, respectively. It is noteworthy that more than half of the cotton CEP genes are located within gene clusters, which are defined as regions containing more than three genes in close proximity. For example, in *G. barbadense*, there are six gene clusters (containing 32 genes) located at the ends of three pairs of homologous chromosomes: A01/D01, A02/D03 and A05/D05.

**Figure 4:**
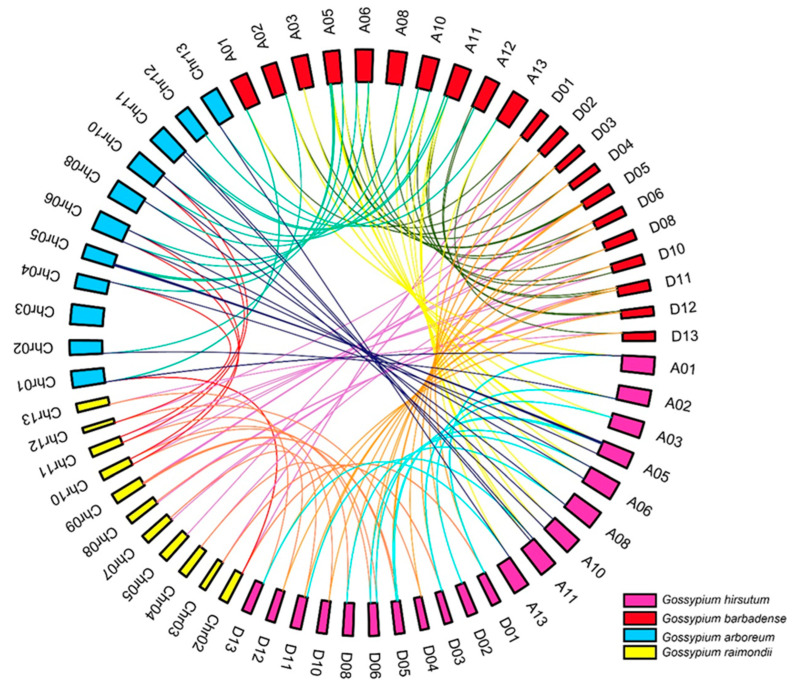
Collinear correlations between CEP genes from Ga, Gr, Gh and Gb. Homologous relationships of CEP genes are indicated with colour lines.

The possible ancestors of *G. hirsutum* and *G. barbadense* are widely considered to be the two extant diploid cotton species, *G. arboretum* and *G. raimondii* [22]. To explore the evolutionary history of the *CEP* gene family in *Gossypium*, synteny analyses were performed to identify directly homologous *CEP* genes in the four cotton species (Figure 4). Collinearity analyses identified 20 directly homologous gene pairs between the D subgenome of *G. hirsutum* and *G. raimondii*, 21 directly homologous gene pairs between the A subgenome of *G. hirsutum* and *G. arboreum*, 11 directly homologous gene pairs between *G. raimondii* and *G. arboreum*, and 16 directly homologous gene pairs between the A and D subgenomes of *G. hirsutum*. These results indicate that the *CEP* family genes have been highly conserved during the evolution of cotton.

### 2.4. Analysis of the Expression Pattern of CEP Gene in G. hirsutum

**Figure 5:**
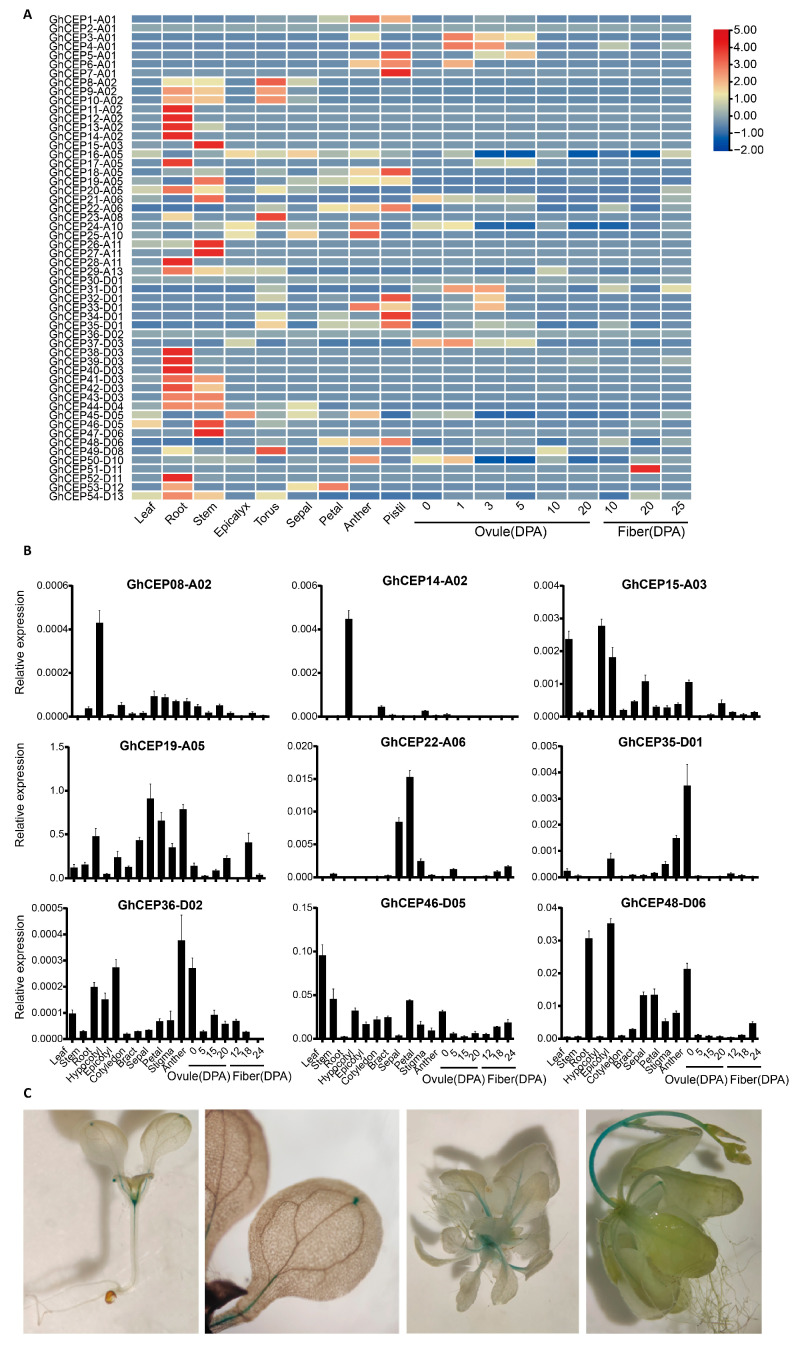
The tissue-specific expression patterns of the CEP family genes in upland cotton (Gh). (**A**) Heat map of expression profiles of *G. hirsutum* CEP genes in public RNA-seq data. (**B**) Expression levels of nine CEP genes in quantitative RT-PCR. (**C**) Expression of *GhCEP46-D05* in *Arabidopsis* detected by GUS histochemical staining. DPA, days after anthesis.

The public RNA-seq data in *G. hirsutum* show that CEP genes in the same gene cluster tend to have the same expression pattern (Figure 5A). For example, the genes in gene clusters on A02/D03 (*GhCEP08-A02~GhCEP14-A02* and *GhCEP38-D03~GHCEP43-D03*) are almost all preferentially expressed in the root. The genes in another pair of gene clusters on A01/D01 (*GhCEP5-A01~GhCEP7-A01* and *GhCEP32-D01~GHCEP35-D01*) are preferentially expressed in the pistil. In addition, several CEP genes show high expression in the stem (*GhCEP15-A03*, *GhCEP26-A11*, *GhCEP27-A11*, *GhCEP46-D05* and *GhCEP47-D06*). No CEP gene is preferentially expressed in fiber except for *CEP51-D11*.

Quantitative RT-PCR was used to further analyse the expression pattern of nine CEP genes. (Figure 5B). Consistent with the RNA-seq data, *GhCEP08-A02* and *GhCEP14-A02* were found to be preferentially expressed in roots, and *GhCEP19-A05* was highly expressed in floral tissues, such as bract, sepal and anther. *GhCEP46-D05* showed a high level of expression in the leaf and stem. Some discrepancies were found between RNA-seq and quantitative RT-PCR for some tissues or CEP genes, which could be due to experimental conditions or differences in the tissues collected.

*GhCEP46-D05* was selected for further analysis of the expression pattern using promoter::GUS reporter lines (Figure 5C). Its expression was found in the shoot apex, leaf veins, leaf tips and stems.

### 2.5. Function of Cotton CEPs in Plant Development

**Figure 6:**
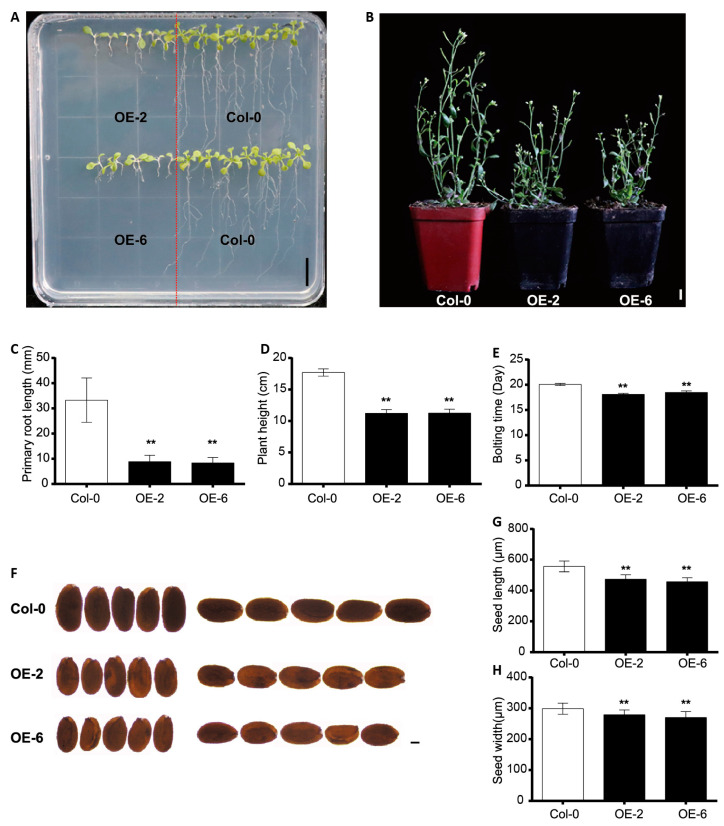
Overexpression of *GhCEP46-D05* in *Arabidopsis.* (**A**) Phenotypes of root. (**B**) Phenotypes of shoot. (**C**–**E**) Quantification of primary root length, plant height and bolting time. (**F**) Phenotypes of seed. (**G**,**H**) Quantification of the seed length and seed width. Data are displayed as mean ± standard deviation; *p*-values are determined by means of Student’s *t*-test (** *p* < 0.01). Scale bars: 1 cm in **A** and **B**; 100 µm in **F**.

To investigate the function of cotton CEP genes in plant growth and development, the *GhCEP46-D05* gene was isolated and subsequently inserted into a construct downstream of the 35S promoter for overexpression. (*p35S*::*GhCEP46-D05*) in *Arabidopsis* Col-0. When grown on standard medium, a strong reduction in primary root length was observed in *p35S::GhCEP46-D05* lines. The final plant height of these overexpression lines was also significantly reduced when compared with Col-0 (Figure 6A–D). These lines also showed an earlier bolting and flowering time (Figure 6E). In addition, both the length and width of mature seeds were reduced in the overexpression lines (Figure 6F–H). 

**Figure 7:**
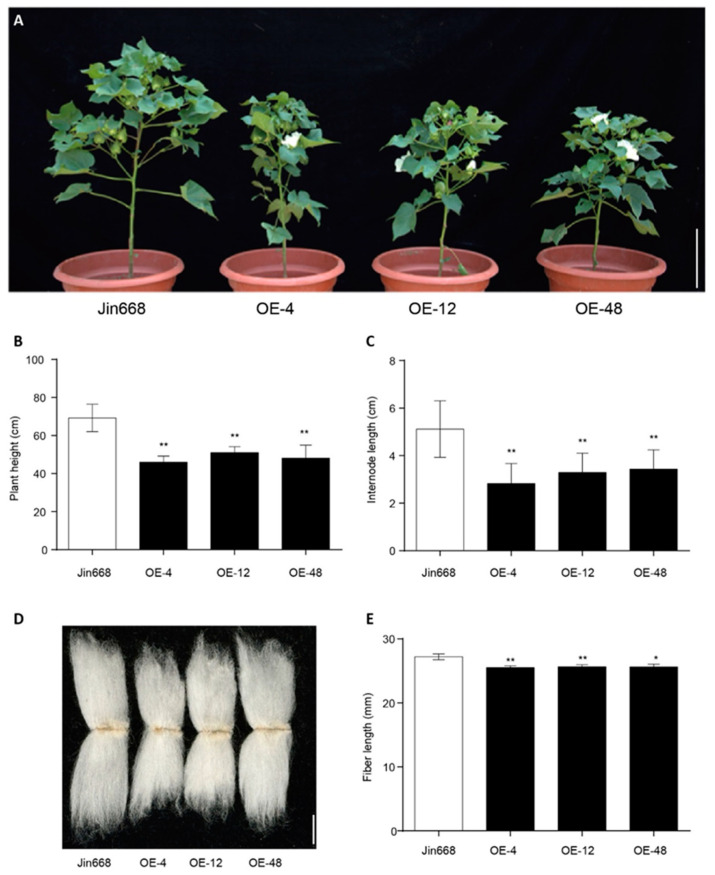
Overexpression of *GhCEP46-D05* in cotton. (**A**) Representative plants of Jin668 and overexpression lines. (**B**,**C**) Quantification of plant height and internode length. (**D**) Phenotypes of fiber. (**E**) Quantification of fiber length. Data are displayed as mean ± standard deviation; *p*-values are determined by means of Student’s *t*-test (** *p* < 0.01, * *p* < 0.05). Scale bars: 10 cm in (**A**) and 10 mm in (**D**).

To further investigate the function of *GhCEP46-D05* in cotton, *p35S::GhCEP46-D05* was also transformed into the cotton line Jin668. Similar to the phenotype in *Arabidopsis*, the overexpression lines showed a reduced plant height resulting from a reduced internode length of the main shoot (Figure 7A–C). In addition, a shorter fiber length was observed in the overexpression lines (Figure 7D,E), suggesting that *GhCEP46-D05* inhibits cell expansion, considering that each fiber is a single cell.

Thus, the above results indicate that the overexpression of GhCEP46-D05 has an effect on the development of roots, shoots and seeds in plants.

## 3. Discussion

Previous studies have identified over 900 putative CEP genes in plant genomes [4,14,23,24]. Despite this, a thorough investigation of the CEP gene family in cotton species has not been undertaken. In our research, we carried out a comprehensive investigation of the cotton CEP genes, covering aspects such as their evolutionary relationships, structural domains, genomic locations, gene synteny and expression patterns.

The two allotetraploid cotton species, *G. hirsutum* and *G. barbadense*, are thought to be derived from natural interspecific hybridization events between their ancestral species, usually speculated to be *G. raimondii* and *G. arboreum* [25]. It would therefore be expected that the total number of CEPs in these two allotetraploid cotton species would be equal to the combined CEPs of *G. raimondii* and *G. arboreum*. However, the CEPs identified in *G. hirsutum* (54) and *G. barbadense* (59) were fewer than the combined CEPs found in the two allotetraploid progenitors, *G. arboreum* (34) and *G. raimondii* (35). This discrepancy suggests that the process of polyploidisation, characterised by chromosome duplication and rapid genome rearrangement, may lead to different levels of gene loss or pseudogenization [26].

A characteristic feature of the CEP genes found in the four cotton species is that many of these genes are located in gene clusters. In Arabidopsis, *AtCEP5*, *AtCEP6*, *AtCEP7*, and *AtCEP8* also appear to be tandem repeats on chromosome 5 [7,8]. This clustered distribution may therefore be a typical feature of the CEP gene family. Compared to *Arabidopsis*, the discovery of a greater number of gene clusters in cotton suggests that tandem gene duplication may be a driving force for the expansion of the cotton CEP gene family. In addition, in this study, we found that the CEP genes in the same cluster tend to have similar expression patterns, and this phenomenon has been found in many previous studies, demonstrating an efficient mechanism for gene replication and expression regulation [27,28].

Phylogenetic tree analysis showed that cotton CEP genes could be separated into two distinct groups (Group I and Group II), which is consistent with the phylogenetic relationships found in *Arabidopsis* and other plants [7,8,12,14,22]. In addition, within each group, the present study found three subgroups that have not been reported in other plants. In Group 1, there are 12 Arabidopsis CEPs (*AtCEP1*–*AtCEP12*), but they are only present in subgroup I-2. This result suggests that the differentiation of novel CEP genes (paralogs) in cotton is another driving force for the expansion of the cotton CEP gene family. Considering that the same conserved CEP domain is shared between these subgroups in each group, it can be hypothesized that the differentiation of non-conserved segments in CEPs is responsible for the formation of subgroups. These subgroups also suggest that there may be functional differentiation within each group of cotton CEPs.

In *Arabidopsis*, CEP gene expression is detected in different tissues at different stages of the plant’s life cycle. Some of these expressions are triggered by different environmental cues, such as abiotic stress, nutrient deficiency, and unfavourable temperature fluctuations. Our results indicate that the expression of cotton CEP genes is widespread throughout the cotton plant, although a subset of these genes have distinct expression profiles. For example, certain genes, such as *GhCEP08-A02* and *GhCEP14-A02*, are more highly expressed in roots, whereas others, such as *GhCEP19-A05* and *GhCEP22-A06*, are highly expressed in flower tissues. These findings suggest that cotton CEP genes play a significant role in the growth and development of cotton plants.

Previous studies have highlighted the important role of CEP genes in various plant growth and developmental processes [5,6,15,29]. In the case of *Arabidopsis*, the enhanced expression of *AtCEP1* or *AtCEP3* has been shown to suppress primary root growth and lateral root emergence [30]. Similarly, in *Medicago truncatula*, overexpression of *MtCEP1* reduces the number of lateral roots and increases the number of nodules [20]. In rice, the upregulation of *OsCEP6.1* resulted in pleiotropic effects on panicle and grain development, such as dwarfism, smaller leaves, fewer tillers, reduced grain size, and fewer grains per panicle. In this study, we found that overexpression of *GhCEP46-D05* in Arabidopsis inhibited primary root elongation and reduced plant height and seed size. In cotton, overexpression of *GhCEP46-D05* also leads to a reduction in internode length and fiber length. This suggests that *GhCEP46-D05* may play a role in the regulation of cotton plant growth. It is possible that *GhCEP46-D05* inhibits internode elongation and fiber growth by decreasing cell elongation. Based on our results, it can be inferred that cotton CEP genes play a crucial role in cotton plant development. This is supported by the observation that *GhCEP46-D05* has similar effects on plant development as observed in other plants such as *Arabidopsis* and rice. Therefore, the present results suggest that manipulation of CEP genes could be an effective way to improve the yield or quality of cotton.

Recently, the CEP signalling pathway has attracted a lot of research attention, and many key factors have been identified, such as the CEP receptors CEPR1, CEPR2 and CRA2 [16,19,20]. Crosstalk between CEP signalling and other hormones, such as ABA and ethylene, has also been observed [17,18,20]. Future studies are needed to identify the CEP receptor genes and other factors involved in CEP signalling and to analyse their functions in plant development or stress resistance in cotton.

## 4. Materials and Methods

### 4.1. Identification of CEP Protein Family Members 

The complete genome sequence data of four cotton species, *Gossypium arboreum* (A2) (CRI v1), *G. raimondii* (D5) (JGI v2), *G. hirsutum* (AD1) (ZJU v2.1) and *G. barbadense* (AD2) (ZJU v1.1), were retrieved from the COTTONGEN database (https://www.cottongen.org/, accessed on 15 July 2022) [31]. The complete amino acid sequences of *CEPs* from *Arabidopsis thaliana* were downloaded from TAIR (https://www.arabidopsis.org/, accessed on 18 July 2022). The *AtCEP* protein sequences were used as queries and a genome-wide similarity search was performed using BLASTP to identify the cotton *CEP* gene family with an E-value cut-off of 1. SignalP 5.0 (https://services.healthtech.dtu.dk/service.php?SignalP-5.0, accessed on 20 July 2022) was used to search for signal peptides. Only sequences containing both the signal peptides and the conserved 15-amino acid peptide (CEP) domains were retained for further analysis [32]. The physicochemical properties of CEP proteins, such as length, molecular weight, and isoelectric point, were predicted using the ExPASy suite of tools (https://www.expasy.org/, accessed on 25 July 2022) [33]. 

### 4.2. Sequence Alignment and Phylogenetic Tree Construction

The Clustal W 1.81 program (http://www.clustal.org/clustal2/, accessed on 12 May 2020) with default parameters was used to align the amino acid sequences of all CEP proteins. The consensus CEP domains of the *G. hirsutum* CEP genes were represented using Weblogo (http://weblogo.berkeley.edu/logo.cgi, accessed on 2 August 2022). The NJ tree with bootstrap tests of 1000 replicates was constructed by aligning the amino acid sequences of the CEP proteins using MEGA X (https://www.megasoftware.net/, accessed on 28 July 2022) [34].

### 4.3. Chromosomes Locations and Collinearity of CEPs in Cotton

The physical location data for the CEP genes were retrieved from the *Gossypium* genomic databases using gff3 files. The local database contained protein sequences from four cotton species, which were searched using the Basic Local Alignment Search Tool (BLAST). The blastp results were analysed using the Dual Systeny Plotter of TBtools and MCscanX to examine the collinearity of the CEP genes [35,36].

### 4.4. Analysis of Expression Pattern of CEPs

To characterize the gene expression patterns of the CEP gene family in different tissues, we downloaded public RNA-seq data from the Cotton Omics Database project website (http://cotton.zju.edu.cn/, accessed on 10 August 2022) [37,38], and the heat map of the tissue expression of the CEP gene family was constructed using the Fragments Per Kilobase of exon model per Million mapped reads (FPKM) values.

### 4.5. RNA Extraction and Quantitative RT-PCR

The Fastpure Plant Total RNA Isolation Kit (Vazyme, Nanjing, China) was used to extract total RNA from various samples (Vazyme, Nanjing, China). First-strand cDNA was synthesized using the PrimeScript RT reagent kit with the gDNA Eraser (Takara, Dalian, China). Quantitative reverse transcription PCR (qRT-PCR) was conducted on a BIO-RAD CFX96 Connect Real-Time PCR System using a TB Green Premix Ex Taq (Tli RnaseH Plus) Kit (Takara, Dalian, China). Relative gene expression level was calculated using the 2^−ΔCt^ method with cotton *ACTIN14* (GeneBank accession number: AY305733) as the internal reference. Three independent biological replicates and four technical replicates were performed for each sample. Primers used are listed in Table S2. 

### 4.6. Promoter Activity Analysis

The 2000-bp upstream fragment of *GhCEP46-D05* was cloned and inserted upstream of the GUS reporter gene in plasmid *pBI121*. The resulting construct was then introduced into *Agrobacterium tumefaciens* strain *GV3101*. *Arabidopsis* ecotype Col-0 was used as the transgenic receptor. Promoter activity analysis was detected by GUS histochemical staining.

### 4.7. Over-Expression Constructs and Plant Transformation

The full-length coding sequence of *GhCEP46-D05* was PCR amplified from the cDNA of TM-1 and then put downstream of the 35S promoter of plasmid *pBI121*. The resulting construct was then transformed into the *Agrobacterium tumefaciens* strain *GV3101*. *Arabidopsis thaliana* was transformed via *Agrobacterium*-mediated transformation using the floral-dip method [39]. Jin668, an upland cotton cultivar with a normal growth habit, was used as the receptor for cotton transformation. The *Agrobacterium*-mediated transformation was carried out according to a previous study [40]. For both *Arabidopsis* and cotton, positive transformants were selected by kanamycin resistance and PCR analysis. The expression of *GhCEP46-D05* was detected by qRT-PCR in the leaves of the transgenic plants.

### 4.8. Phenotypic Analysis of GhCEP46-D05 Transgenic Plants

For *Arabidopsis*, seeds from Col-0 (wild-type, WT) and transgenic plants (T3 generation) overexpressing *GhCEP46-D05* were germinated on Murashige and Skoog (MS) medium containing 3% sucrose and 0.7% agar (pH 5.8), and Petri dishes were placed vertically to allow the roots of the plants to grow downwards. After vernalization, the seeds were incubated at 21 °C with a 16:8 h light/dark photoperiod. After seven days, 30 plants from each line were randomly selected for the measurement of primary root length. After 15 days, the seedlings were transferred to soil and grown in a growth chamber (at 21 °C with a 16:8 h light/dark photoperiod). The bolting date was defined as the number of days between seed germination and the time when flower buds could be distinguished from leaves. Plant height was measured 40 days after sowing. Seed length and width were measured using ImageJ software (https://imagej.net/software/imagej/, accessed on 8 March 2019). For cotton, seeds were germinated at 28 °C with a 16 h light/8 h dark cycle on 1/2X MS medium, and then seedlings were transplanted into the soil to grow to maturity under normal growing conditions. For each line, 20 plants were randomly selected for measurement of plant height and main shoot internode length at maturity. Mature fibers (15 g/each sample) collected from the bolls at similar positions on the plants (at least 15 plants per line) were used for fiber length measurements using an HVI900 instrument (USTER, Knoxville, TN, USA) at the Center of Cotton Fiber Quality Inspection and Testing, Chinese Ministry of Agriculture (Anyang, China). Statistically significant differences between groups were determined using the Student’s *t*-test with GraphPad Prism 8.0 software.

## 5. Conclusions

CEPs are a group of widely distributed peptide hormones in plants. Although numerous CEP genes have been identified in different plants, their functions remain largely unknown, particularly in cotton. In this study, we performed a comprehensive analysis of CEP gene families in four cotton species for the first time. A total of 182 CEP genes were identified on the basis of their conserved CEP domains and the presence of a signal peptide. The cotton CEPs were divided into two groups, each with three subgroups. Further analysis was conducted on the conserved motifs, chromosomal locations, gene collinearity, and expression patterns. The results suggest that segmental duplications may have a significant impact on the expansion of cotton CEPs, with more than half of them located in gene clusters. The CEP domains in both groups showed a high degree of similarity, with a core motif present in both. Expression pattern analysis revealed that CEP genes within the same gene cluster tended to exhibit the same pattern. Expression analyses showed that some CEP genes are expressed exclusively in certain tissues, such as roots and pistils. An additional functional analysis was performed on *GhCEP46-D05*. Its overexpression inhibits root, shoot and fiber elongation, probably by repressing cell expansion. The results show that the CEP genes in cotton play a key role in plant development. This study provides a solid basis for further investigation of CEP gene functions in cotton.

## Data Availability

Data are contained within the article or Supplementary Materials.

## Supplementary Materials

The following supporting information can be downloaded at: https://www.mdpi.com/article/10.3390/ijms25084231/s1.
